# Editorial: Reviews in pulmonary medicine 2022

**DOI:** 10.3389/fmed.2023.1296581

**Published:** 2023-12-04

**Authors:** Shuibang Wang, Bruno Guedes Baldi

**Affiliations:** ^1^Critical Care Medicine Department, Clinical Center, National Institutes of Health, Bethesda, MD, United States; ^2^Pulmonary Division, Heart Institute (InCor), University of São Paulo Medical School, São Paulo, Brazil

**Keywords:** lung disease, lung cancer, chronic obstructive pulmonary disease (COPD), ARDS, cell senescence and apoptosis, lymphangioleiomyomatosis (LAM)

Lung diseases are a major cause of morbidity and mortality worldwide. In recent years, there have been significant advances in our understanding of the mechanisms of lung diseases, as well as the development of new treatments. The collection of *Reviews in Pulmonary Medicine 2022* reviewed several lung diseases and their treatments.

Sepsis-related acute respiratory distress syndrome (ARDS) is a life-threatening condition that occurs when inflammation in the lungs leads to fluid buildup and breathing problems. It is a major cause of death in sepsis patients, with a fatality rate of 30–40% (Gong et al.). Currently, there is no cure for ARDS. The main treatment is supportive care, such as mechanical ventilation and fluid management. However, there are ongoing research efforts to develop new treatments that target the underlying mechanisms of ARDS. Some of the potential targets for ARDS treatment include inflammatory mediators, such as cytokines and chemokines, endothelial cell dysfunction, epithelial cell injury, disruption of VE-cadherin, alveolar macrophage activation, neutrophil apoptosis, and excessive production of reactive oxygen species (ROS) (Gong et al.). To gain new insights into ARDS pathogenesis and to identify and develop new therapeutic targets, biomarkers of the disease have been actively sought. These biomarkers, such as the soluble form of the receptor for advanced glycation end-products (sRAGE) and angiopoietin-2 (ANG2), are believed to be markers of type I alveolar epithelial cell injury and lung endothelial barrier dysfunction, respectively. High levels of sRAGE and ANG2 are also associated with an increased risk for ARDS and severity of the disease. The precise role of sRAGE in ARDS remains a topic of ongoing debate. A prevailing view suggests that sRAGE plays a protective anti-inflammatory role by acting as a decoy receptor, preventing RAGE ligands from binding to membrane-bound RAGE. However, opposing arguments propose that elevated sRAGE levels may instead result from overstimulation of cell surface RAGE, leading to downstream inflammatory signaling (1). In contrast, elevated ANG2 levels are believed to directly contribute to the increased risk of developing ARDS (2). Experimental evidence suggests that reducing blood ANG2 or blocking its signaling can improve survival in animal models (3).

Chronic obstructive pulmonary disease (COPD) is the third leading cause of death worldwide. It is characterized by the destruction of the airways and alveoli and caused by smoking and air pollution. COPD is incurable, and its pathogenesis is complex and not fully understood. Multiple factors and their interactions, including persistent inflammation, oxidative stress, and protease/antiprotease imbalance, are known factors contributing to the pathogenesis of COPD. In addition, most recent studies suggest that ferroptosis also plays an important role in the pathogenesis of COPD, and it may be a target for new therapies (Meng et al.). Different from apoptosis and necroptosis, ferroptosis is a recently discovered form of cell death that is mediated by phospholipid peroxidation through free iron-mediated Fenton reactions in the presence of ROS. Cigarette smoke exposure can induce ferroptosis in lung epithelial cells by increasing the levels of iron and ROS in the lungs, leading to cell death and tissue damage. Ferroptosis can also be triggered by the depletion of glutathione (GSH), an antioxidant that protects cells from ROS damage and detoxifies lipid peroxides. Deficiency or inhibition of *SLC7A11* (solute carrier family 7 member 11), *GPX4* (glutathione peroxidase 4), and *NFE2L2* (NFE2 like BZIP transcription factor 2, also known as *NRF2*) genes, which direct generation of GSH, superoxide dismutase, catalase, heme oxygenase, and other antioxidants, exacerbate ferroptosis in COPD. Therefore, targeting ferroptosis by inducing these genes and/or by removing excessive iron from the body may be a promising new approach for the treatment of COPD.

Lung cancer (LC) is the leading cause of cancer death worldwide, with about 1.8 million deaths in 2020 as estimated by the International Agency for Research on Cancer. Therapies such as chemotherapy, radiation, and their combination, can be effective at initial treatment of LC, but they often have relapse of the disease that then manifests in a chemotherapy-resistant form. In recent years, there has been a growing interest in a new approach to LC therapy called immunogenic cell death (ICD) (Xu et al.). ICD is a form of regulated cell death in which damage-associated molecular patterns (DAMPs) and tumor-associated antigens (TAAs) are released. These molecules activate dendritic cells to present tumor antigens to T cells, which then kill the LC cells. In this way, the dying cancer cells are transformed into a therapeutic vaccine that can boost the body's defenses specifically against LC and provide long-term effects to the patients. To date, the DAMPs that have been mechanistically linked to ICD include ATP, CALR (calreticulin), HMGB1 (high mobility group box 1), Type I IFN (interferon), and ANXA1 (annexin A1). Since doxorubicin was identified as the first ICD inducer in 2005 (4), different therapeutic agents have been found to initiate ICD in LC. For example, crizotinib, a targeted therapy used to treat LC patients with *ALK* (ALK receptor tyrosine kinase) gene rearrangements, can induce ICD in *ALK*-positive LC cells. Lurbinectedin, another potent ICD inducer, was approved by the US Federal Drug Administration in 2020 for the treatment of relapsed small-cell LC (5). To kill cancer cells more effectively and achieve better results of LC treatment, different ICD inducers are being used or are on clinical trials in combination with immune checkpoint inhibitors, such as pembrolizumab, which targets PD-1 (programmed death protein 1)/PD-L1 (programmed death ligand 1) pathway (5).

In addition to sepsis-related ARDS, COPD, and LC, the collection of reviews also includes other pulmonary diseases, such as interstitial lung diseases (ILDs), including rare lymphangioleiomyomatosis (LAM), and cryptogenic organizing pneumonia (COP), portopulmonary hypertension (PoPH), eosinophilic granulomatosis with polyangiitis (EGPA), pleural effusion, and obstructive sleep apnea. Clinical features, pathogenesis, diagnosis, current treatment options and future perspectives for these diseases have been discussed in detail. For instance, research has discovered that LAM is linked to mutations in the *TSC2* or *TSC1* gene, which trigger hyperactivation of the mTORC1 signaling pathway and subsequent LAM cell senescence (Bernardelli et al.). Cellular senescence, characterized by a permanent proliferation arrest, anti-apoptosis, and proinflammatory phenotype, is also observed in COPD (6), LC (7), idiopathic pulmonary fibrosis (8), and pulmonary arterial hypertension (9). Therefore, hindering senescence and eliminating senescent cells using mTORC1 inhibitors and senolytic drugs could be potential therapeutic strategies for LAM and these lung diseases.

In summary, lung diseases continue to be a significant global health concern. Researchers have been focusing on the discovery of genetic susceptibility, the molecular mechanism of disease, various biomarkers, immune dysfunction, and environmental/lifestyle triggers. Advancements in these research areas have facilitated the development of targeted therapies, early diagnosis and disease monitoring, personalized treatment, and potential preventive strategies. Gene therapy and gene editing, though still in the early stages of development, hold promise for correcting genetic defects that contribute to lung diseases. Mesenchymal stem cell-based therapies could also offer the possibility of regenerating damaged lung tissue and restoring lung function. Additionally, artificial intelligence and machine learning tools can enable researchers and clinicians to analyze vast amounts of data, process images, and identify patterns and associations, thereby accelerating progress in these research efforts.

## Author's note

The opinions expressed in this article are the authors' own and do not represent any position or policy of the National Institutes of Health, the Department of Health and Human Services, or the United States Government.

## Author contributions

SW: Writing – original draft, Writing – review & editing. BB: Writing – original draft, Writing – review & editing.
